# Sizing up Testicular Masses: Clinical Implications of Lesion Size and Contralateral Biopsy

**DOI:** 10.3390/cancers18182907

**Published:** 2026-09-08

**Authors:** Elena Katharina Berg, Gwendolin Seidel, Lennert Eismann, Yannic Volz, Paulo Leonardo Pfitzinger, Philipp Weinhold, Gerald Bastian Schulz, Patrick Keller, Isabel Katharina Brinkmann, Fabian Horné, Christian Georg Stief, Julian Marcon, Robert Bischoff

**Affiliations:** 1 Department of Urology, LMU University Hospital, LMU Munich, Marchioninistrasse 15, 81377 Munich, Germany; 2 Division of Cancer Surgery, Peter MacCallum Cancer Centre, 305 Grattan Street, Melbourne 3000, Australia; 3 Department of Urology, TUM University Hospital, TU Munich, Ismaninger Strasse 22, 81675 Munich, Germany

**Keywords:** germ cell tumor, testicular cancer, sonography, lesion size, biopsy, orchidectomy

## Abstract

**Background/Objectives**: Accurate preoperative discrimination between benign and malignant testicular masses remains challenging. Lesion size has been suggested as a predictor, but robust cutoffs and the role of contralateral biopsy remain uncertain. **Methods**: We retrospectively analyzed a prospectively maintained single-center database of 387 patients treated for testicular lesions between December 2013 and November 2024. Patients with malignant non-germ cell histologies or prior systemic GCT therapy were excluded. Lesion size was evaluated as a predictor of malignancy using ROC analysis, logistic regression, and predefined cutoffs (5, 8, 10, 15 mm). Associations between contralateral biopsy results and clinical variables were assessed using uni- and multivariable analyses. **Results**: Of 387 patients, 284 (73.4%) had malignant GCTs. Malignant lesions were significantly larger than benign ones (median 25.0 vs. 5.5 mm, *p* < 0.001). Lesion size showed good discrimination between benign and malignant lesions (AUC 0.829, 95% CI 0.784–0.874), with a Youden-derived optimal cutoff of 12.5 mm. Internal bootstrap validation (2000 resamples) yielded a median optimal cutoff of 12.5 mm (95% percentile interval 7.45–19.5 mm), indicating uncertainty regarding the exact threshold. Among 360 patients with complete clinical and tumor-marker data, combining lesion size with age, AFP, β-HCG, and LDH significantly improved discrimination compared with lesion size alone (AUC 0.863 vs. 0.818; DeLong *p* = 0.003). Contralateral biopsy was performed in 249 patients, with 7 (2.8%) positive for GCNIS. Younger age was associated with contralateral GCNIS, while tumor size, serum markers, and histology were not predictive. Multivariable analysis showed no significant predictors. **Conclusions**: Lesion size is a strong predictor of malignant histology, although the optimal size threshold remains subject to uncertainty. Combining lesion size with readily available clinical and biochemical parameters may further improve preoperative risk stratification. Contralateral GCNIS was rare and not reliably predicted, supporting individualized biopsy strategies.

## Simple Summary

Distinguishing harmless from cancerous testicular masses before surgery remains difficult and often leads to uncertainty in treatment decisions. In this study, we analyzed 387 patients to determine whether the size of a testicular mass can help predict the likelihood of cancer and whether a biopsy of the opposite testicle can be guided by clinical features. We found that larger masses were much more likely to be cancerous, with masses larger than 15 mm carrying a very high risk of malignancy. Precancerous changes in the opposite testicle were uncommon and could not be reliably predicted using routine clinical factors. These findings provide practical size thresholds that may improve preoperative counselling and risk assessment and further support an individualized approach to biopsy of the opposite testicle rather than routine testing.

## 1. Introduction

Testicular cancer represents the most common solid cancer entity in young males and accounts for about 5% of all urological tumors [1]. About 95% of these cases are germ cell tumors (GCTs), which are classified into seminomatous and non-seminomatous germ cell tumors (NSGCTs) based on histopathological features and clinical behavior [2,3]. Early detection and accurate risk stratification are essential, as appropriate management results in excellent long-term survival for most patients [2,4]. Patients typically present with a painless, palpable, indurated scrotal mass of variable size. Consequently, scrotal sonography remains the imaging modality of choice for detailed local assessment, lesion characterization and accurate size measurement. With the widespread use of ultrasound, the detection rate of testicular masses (STMs) has increased substantially over recent decades [5]. When a testicular lesion is considered suspicious, surgical exploration via an inguinal approach remains the standard of care [6]. However, a considerable proportion of orchiectomy specimens reveal benign findings, including Leydig cell tumors, granulosa cell tumors, cysts, hematomas or areas of necrosis—lesions that would not have required orchiectomy in retrospect [7]. An organ-sparing approach with frozen section examination can alternatively be considered in selected cases, particularly for small or indeterminate lesions [8,9,10,11]. Although sonographic features such as inhomogeneous echogenicity, increased vascularization, and ill-defined margins have been associated with malignancy, reliable preoperative differentiation between benign and malignant lesions remains challenging [5,12,13,14,15]. The association between lesion size and histopathology is not well established, and no validated size threshold exists. With the rising incidence of STMs and the potential for overtreatment, including infertility and endocrine dysfunction, improved preoperative diagnostic tools are needed to better distinguish malignant from benign lesions and reduce unnecessary orchiectomies.

The risk of contralateral germ cell neoplasia in situ (GCNIS) in patients with unilateral GCT ranges from approximately 4% to 8% and is higher in individuals with known risk factors such as cryptorchidism and testicular atrophy [16,17,18,19]. Advances in contralateral biopsy protocols, particularly the use of immunohistochemical markers, have improved GCNIS detection and reduced the incidence of metachronous tumors. However, routine contralateral biopsy remains controversial, and current guidelines recommend its use primarily for high-risk patients [17,20,21,22].

The present study aimed to investigate the association between sonographic lesion size and likelihood of malignancy in testicular masses. By correlating imaging findings with histopathological results, we sought to determine whether lesion size can serve as a reliable predictor of malignancy and thereby improve diagnostic stratification. Additionally, we evaluated the utility of contralateral biopsies and explored potential predictive factors for contralateral biopsy positivity in a real-world clinical cohort.

## 2. Materials and Methods

### 2.1. Patient Cohort and Study Design

In the present study, we retrospectively analyzed data from our prospectively maintained institutional testicular tumor database. All consecutive patients presenting with a testicular mass who underwent either surgical biopsy, enucleation, or radical orchiectomy at our tertiary care urology center between January 2014 and November 2024 were eligible for inclusion. At first presentation, all patients underwent routine physical examination and sonographic assessment in supine position using a linear transducer, including testicular B-mode ultrasound. Ultrasound examinations were mainly carried out by urologists in the outpatient clinic or ward, while a subset of patients underwent contrast-enhanced ultrasound performed by a radiologist at the local radiology department. Lesion size was defined as the maximum lesion diameter measured on preoperative ultrasound. Examinations were performed by different operators as part of routine clinical care, and no formal assessment of interobserver variability was available. Clinical, pathological, and perioperative variables were retrieved from electronic health records and cross-checked against operative notes and histopathology reports. Patients with malignant histologies other than germ cell tumors or metastatic lesions from extratesticular primaries, as well as those who had previously received systemic therapy for germ cell tumors, were excluded. In cases with multiple testicular lesions, the first or dominant lesion was considered for analysis. Contralateral biopsy was not routinely performed in all patients but was individualized according to contemporary guideline recommendations and clinical judgment, considering established risk factors such as testicular atrophy, previous cryptorchidism and impaired fertility.

### 2.2. Ethics Approval Statement

All study procedures and data collection complied with the principles of the Declaration of Helsinki and were approved by the institutional ethics committee (approval no. 25-0976).

### 2.3. Statistical Analysis and Outcomes

The primary objective was to evaluate the association between tumor size and the probability of histologically confirmed malignancy. Secondary objectives included assessing whether primary tumor size correlated with the risk of contralateral germ cell neoplasia in situ (GCNIS) in patients who underwent contralateral biopsy. As an additional subgroup, small testicular masses were defined as testicular lesions with a diameter of <10 mm.

Continuous variables are reported as median with interquartile range (IQR) and compared between groups using the Wilcoxon rank-sum test. Categorical variables are summarized as frequencies and percentages, and compared using Fisher’s exact test.

To quantify the discriminative ability of tumor size for predicting malignancy, receiver operating characteristic (ROC) analyses were performed. The optimal cutoff value was determined using the Youden index and the area under the curve (AUC) was reported with 95% confidence intervals (CIs). Internal validation of the Youden-derived cutoff was performed using 2000 bootstrap resamples. In each bootstrap sample, the optimal lesion-size threshold was re-estimated using the Youden index. Median bootstrap estimates and percentile-based 95% intervals were calculated for the optimal cutoff, sensitivity, and specificity. Clinical utility was further assessed using decision-curve analysis. Net benefit was evaluated across a range of threshold probabilities for both a continuous logistic regression model based on lesion size and a dichotomized model applying the Youden-derived 12.5-mm threshold. Both strategies were compared with default strategies of treating all and treating none. Logistic regression models were used to explore the odds of malignancy across a range of lesion size thresholds, with results displayed as odds ratios (ORs) and 95% CIs.

To provide a clinically interpretable representation of malignancy probability across the full-size spectrum, we estimated the absolute risk of malignancy by lesion size. First, observed risks were calculated in predefined size bins (0–5 mm, 6–10 mm, 11–15 mm, 16–20 mm, 21–25 mm, 26–30 mm, 31–40 mm, 41–50 mm, and >50 mm). Within each bin, we computed the proportion of malignant histology and corresponding 95% confidence intervals (CIs) using the Wilson method.

In addition, a logistic regression model with natural cubic splines (3 degrees of freedom) was fitted to model malignancy risk continuously across tumor size. Predicted risks with 95% confidence bands were overlaid on the observed bin-level risks to visualize the smoothed relationship between lesion size and malignancy. Clinically relevant thresholds (5, 8, 10, 12.5, and 15 mm, including the Youden-derived cutoff) were annotated in the figure for reference.

Among patients with invasive germ cell tumors, multivariable logistic regression was additionally performed to assess the association between lesion size and lymphatic invasion, vascular invasion, and rete testis involvement, adjusting for age and histologic subtype (seminoma vs. non-seminoma). Lesion size was modeled continuously and odds ratios are reported per 5-mm increase.

To assess whether additional preoperative clinical information improved malignancy prediction beyond lesion size alone, a multivariable logistic regression model incorporating lesion size, age, AFP, β-HCG, and LDH was fitted in patients with complete data. Tumor markers were log-transformed because of their skewed distributions. Discrimination was quantified by the AUC and compared with the size-only model using DeLong‘s test.

For analyses restricted to patients who underwent contralateral testicular biopsy, the outcome of interest was biopsy positivity for GCNIS. Univariable analyses were performed to assess associations between contralateral biopsy outcomes and patient age, an established parameter of risk for contralateral GCNIS, preoperative serum tumor marker levels of alpha-fetoprotein (AFP), beta-human chorionic gonadotropin (β-HCG) and lactate dehydrogenase (LDH), primary tumor size and histology.

For multivariable modeling, patient age, preoperative serum tumor marker levels and primary tumor size were evaluated as predictors. Patient age at diagnosis was entered into the regression models as a continuous variable, scaled per 10-year increase to improve interpretability of effect estimates. Because the number of positive contralateral biopsies was small (*n* = 7), we limited multivariable modeling to these variables to avoid overfitting. To account for potential bias from quasi-complete separation, we applied Firth’s bias-reduced logistic regression using the logistf package in R. Tumor markers were log-transformed to normalize right-skewed distributions, and tumor size was included as a continuous variable. Results are presented as odds ratios (ORs) with 95% confidence intervals (CIs). Model performance was further assessed using the area under the receiver operating characteristic curve (AUC).

All statistical analyses were conducted using R software (version 4.1.0, R Core Team 2021). Two-sided *p*-values <0.05 were considered statistically significant.

## 3. Results

### 3.1. Baseline Characteristics

A total of 387 patients were included in the analysis. The median age at diagnosis was 37 years (IQR 30–46). Malignant GCTs were found in 284 (73.4%) patients. Seminoma represented the most frequent histology (50.6%), followed by benign lesions (26.6%) and non-seminomatous GCTs (22.5%). The individual histopathological diagnoses of the benign lesions are detailed in Supplementary Table S1. The median primary tumor size was 19.0 mm (IQR 7–35). Most tumors were stage pT1 (79.2%), with lymphatic or vascular invasion present in approximately 10% of cases, and rete testis involvement observed in approximately 25%. Serum tumor mar1ker levels were generally within normal or mildly elevated ranges. Contralateral testicular biopsy was performed in 249 patients (64.3%), of whom 7 (2.8%) were positive for GCNIS. Detailed baseline characteristics are summarized in Table 1.

### 3.2. Association Between Tumor Size, Malignancy and GCT Risk Factors

**Figure 1:**
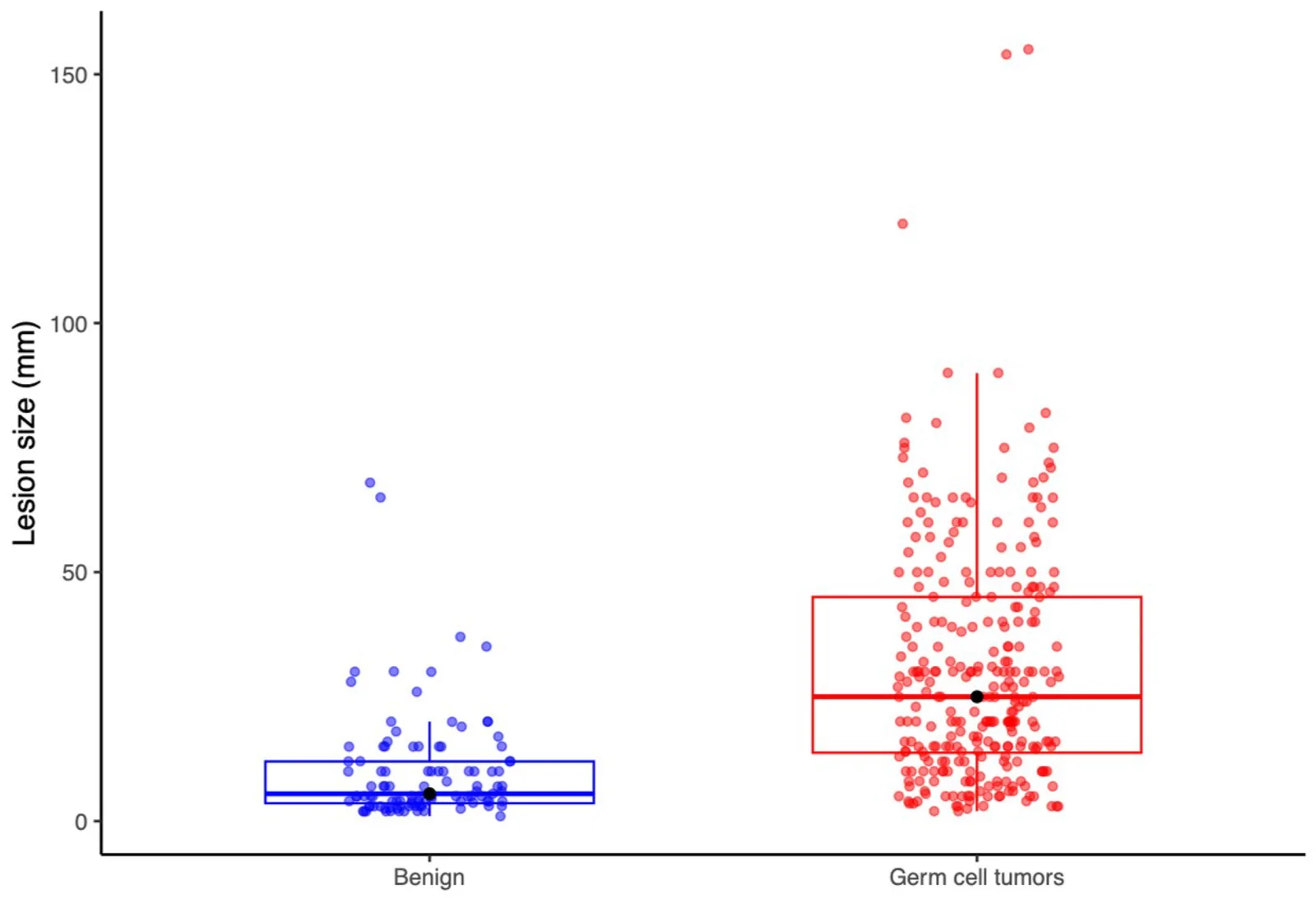
Box plot displaying testicular tumor size by malignancy status.

Tumor size was significantly different between benign lesions and GCTs in the study cohort (*p* < 0.001, Figure 1). In benign lesions, the median tumor size was 5.5 mm (IQR: 3.7–12.0), while GCTs were larger with a median size of 25.0 mm (IQR: 13.8–45.0). Further, tumor size was significantly associated with several risk factors of germ cell tumors: Lymphovascular invasion (*p* < 0.001), vascular invasion (*p* < 0.001) and rete testis infiltration (*p* < 0.001).

In the STM subcohort, a significant association between malignancy and tumor size could also be observed (*p* < 0.001). The size distribution of benign and malignant lesions within the ≤10-mm subgroup is shown in Supplementary Figure S1, demonstrating substantial overlap between bot-h histologic groups across this size range. Median STM size was smaller in benign lesions (4.0 mm, IQR: 3.0–5.0), compared with GCTs (5.0 mm, IQR: 4.0–7.0). Tumor size was significantly associated with lymphovascular invasion (*p* = 0.02) and trended towards significance for vascular invasion (*p* = 0.05). There was no significant association with respect to rete testis infiltration (*p* = 0.33).

### 3.3. Optimal Size Cutoff Values

**Figure 2:**
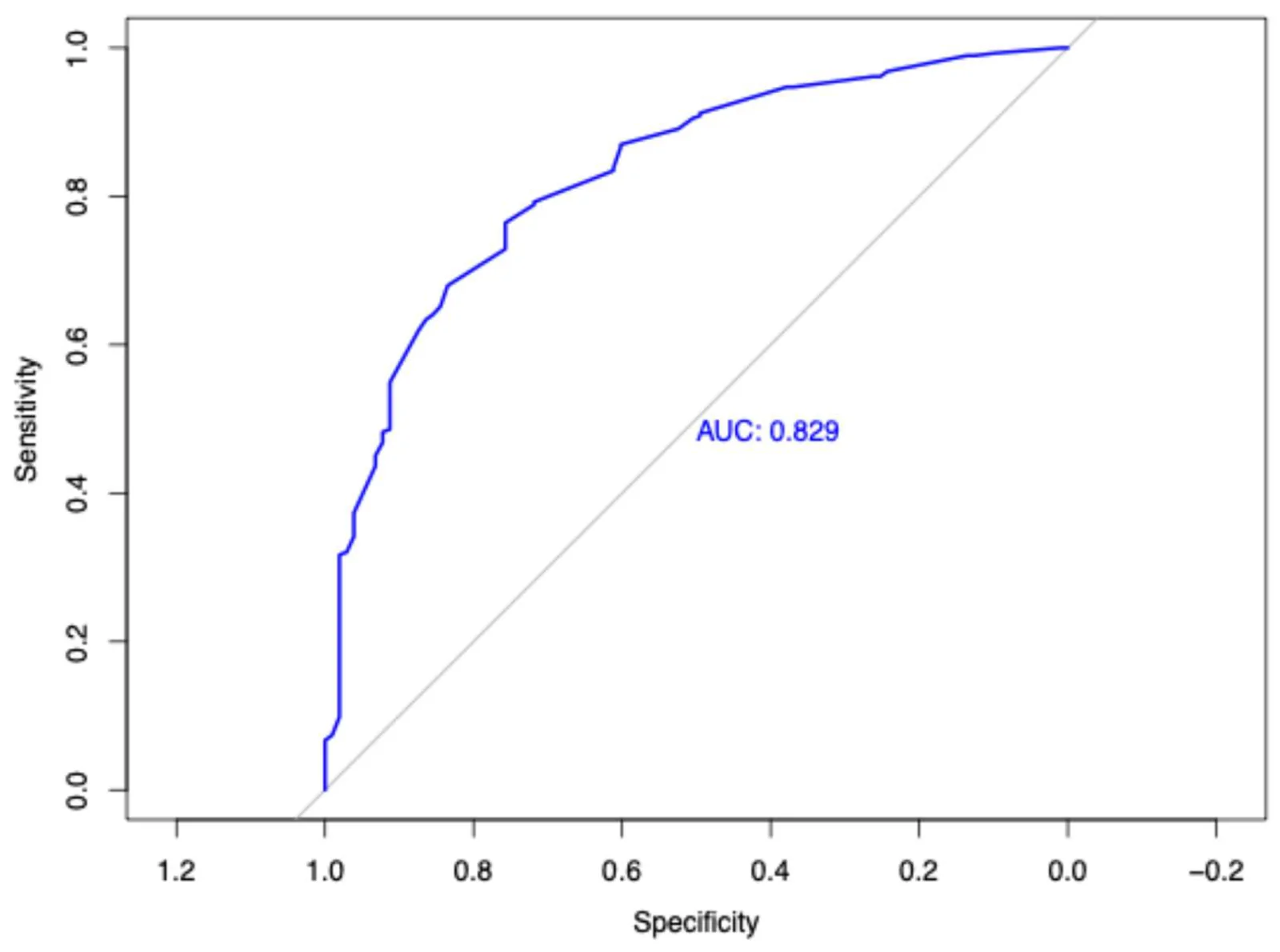
ROC curve displaying the predictive ability of tumor size regarding malignancy in testicular masses.

ROC analysis demonstrated that tumor size discriminated malignant from non-malignant lesions with an AUC of 0.829 (95% CI: 0.784–0.874, Figure 2). The optimal cutoff identified by the Youden index was 12.5 mm, yielding a sensitivity of 76.4%, specificity of 75.7%, and overall accuracy of 76.2% for predicting malignancy. The results of the predefined clinical size thresholds (5, 8, 10, and 15 mm) are displayed in Table 2, illustrating the trade-off between sensitivity and specificity compared with the Youden-derived cutoff at 12.5 mm.

Bootstrap internal validation reproduced the Youden-derived threshold, with a median optimal cutoff of 12.5 mm (95% percentile interval 7.45–19.5 mm). Median bootstrap sensitivity was 75.3% (95% interval 60.4–87.9%) and median specificity was 79.8% (95% interval 65.2–91.7%).

Decision-curve analysis demonstrated a positive net benefit of size-based risk stratification across a broad range of threshold probabilities. The 12.5-mm cutoff provided greater net benefit than the treat-all and treat-none strategies at higher clinically relevant threshold probabilities, while the continuous tumor-size model showed comparable or greater net benefit across most of the evaluated range (Supplementary Figure S2).

### 3.4. Association of Tumor Size with Adverse Pathological Features

Among patients with invasive germ cell tumors, increasing lesion size remained independently associated with lymphatic invasion (OR 1.11 per 5-mm increase, 95% CI 1.03–1.20; *p* = 0.006), vascular invasion (OR 1.17, 95% CI 1.08–1.26; *p* < 0.001), and rete testis involvement (OR 1.13, 95% CI 1.06–1.19; *p* < 0.001) after adjustment for age and histologic subtype.

### 3.5. Malignancy Prediction Using Lesion Size and Clinical Parameters

In a complete-case analysis of 360 patients, the size-only model achieved an AUC of 0.818 (95% CI 0.769–0.867). Incorporating age and preoperative AFP, β-HCG, and LDH increased the AUC to 0.863 (95% CI 0.824–0.902), representing a significant improvement in discrimination (DeLong *p* = 0.003).

### 3.6. Absolute Risk of Malignancy Depending on Tumor Size

The probability of malignancy increased steadily with lesion size (Supplementary Figure S3). In size-stratified analyses, the malignancy rate was lowest in very small lesions (≤5 mm), with risk rising progressively across subsequent size bins.

Spline-based logistic regression demonstrated a continuous increase in the predicted probability of malignancy with increasing lesion size, with the steepest increase observed among smaller lesions and a gradual plateau at larger lesion sizes (Supplementary Figure S4).

Together, these findings support lesion size as a strong predictor of malignant histology, with risk increasing in a dose-dependent fashion and particularly high beyond 15 mm. The detailed results are displayed in Supplementary Table S2.

### 3.7. Predictive Ability of Clinical and Histopathological Parameters Regarding GCNIS in Contralateral Biopsies

Of the 249 patients (64.3%) who underwent contralateral testicular biopsy, 7 (2.8%) were positive for GCNIS. Four of these patients presented with non-seminomatous GCT and three with seminomatous GCT. In univariable analysis, younger patient age, an established risk factor for contralateral GCNIS, was significantly associated with contralateral biopsy positivity (*p* = 0.04).

In univariable analysis, preoperative LDH levels showed a trend toward association with contralateral biopsy positivity (*p* = 0.07), whereas AFP and β-HCG were not significantly associated (*p* = 0.16 and *p* = 0.27, respectively). Tumor size was likewise not associated with contralateral positivity (*p* = 0.14). Histopathological parameters—including T stage, lymphovascular invasion, vascular invasion, and rete testis infiltration—were also not significantly associated with contralateral GCNIS (*p* = 0.19, *p* = 0.50, *p* = 0.08, and *p* = 0.10, respectively).

Multivariable penalized logistic regression was performed among patients who underwent contralateral testicular biopsy to identify independent predictors of contralateral biopsy positivity. Patient age per ten-year increase, tumor size, as well as preoperative serum tumor markers (AFP, β-HCG, and LDH; log-transformed), were included in the model. None of the evaluated variables demonstrated a statistically significant association with contralateral positivity. Tumor size showed no independent predictive value (OR per mm increase 1.03, 95% CI 0.99–1.08; *p* = 0.13). Similarly, AFP, β-HCG, and LDH were not significantly associated with contralateral biopsy outcomes (all *p* > 0.1, Supplementary Table S3). These findings indicate that neither primary tumor burden nor biochemical tumor activity independently predicts the presence of contralateral germ cell neoplasia in situ. Model discrimination was moderate, with an area under the receiver operating characteristic curve (AUC) of approximately 0.84 (Supplementary Figure S5), although confidence intervals were wide, reflecting the low event rate.

## 4. Discussion

Given that GCTs are the most common type of cancer affecting young men, accurate diagnosis and risk stratification are essential. In this single-center retrospective study, we aimed to determine whether lesion size in testicular masses is associated with malignancy. Our cohort reflects the established epidemiology of GCTs, with a median age of 37 years at diagnosis [2]. Malignant GCTs constituted the majority of cases (73.4%), with seminoma being the most frequent histological subtype, consistent with previous studies [23,24]. The median primary tumor size of 19.0 mm and the predominance of pT1 tumors (79.2%) reflect the well-documented observation that most GCTs are diagnosed at an early stage [2,17]. We identified a significant association between lesion size and malignancy, with a median size of 5.5 mm for benign lesions and 25.0 mm for GCTs, respectively. Tumor size was also significantly associated with adverse pathological features, including lymphovascular invasion, vascular invasion, and rete testis infiltration, underscoring its biological relevance. These findings are consistent with the study by Song et al., who reported an increasing risk of malignancy with larger lesion diameters (HR = 1.284 per cm increase, *p* = 0.036). However, their reported median lesion sizes for benign and malignant masses (2.5 mm versus 5.0 mm) were substantially smaller than those observed in our cohort. Similarly, Dieckman et al. demonstrated significant size differences between benign lesions and GCTs (median 10 mm vs. 30 mm) and further showed that other malignant scrotal tumors—predominantly testicular lymphoma—were even larger (median 53 mm), while no size differences were observed between seminomatous and non-seminomatous GCTs [24]. Notably, the proportion of benign lesions in that cohort was relatively low (12.3%). Shilo et al. stratified testicular masses into three size categories (≤10 mm, 11–20 mm and >20 mm) and reported an overall benign rate of only 8%, compared to 26.6% in our cohort. In their study, 50% of lesions <10 mm were benign, which aligns closely with our findings, showing a malignancy rate of 44.4% in lesions ≤10 mm [25]. In our cohort, the lowest malignancy probability was observed in lesions ≤5 mm (32.9%), whereas lesion >15 mm carried a malignancy risk exceeding 90%.

ROC analysis demonstrated that tumor size is a robust discriminator between malignant and benign lesions, with an AUC of 0.829 (95% CI: 0.784–0.874), indicating good diagnostic performance. While lower thresholds (e.g., 5–8 mm) yielded high sensitivity, they resulted in substantial misclassification of benign lesions. A Youden-derived cutoff of 12.5 mm provided the most balanced trade-off between sensitivity (76.4%) and specificity (75.7%), independent of disease prevalence. In comparison, Shilo et al. reported an optimal cutoff of 18.5 mm with higher sensitivity (87%) and specificity (83%) and an AUC 0.902, noting that 38.5% of lesions below this threshold were benign. The intermediate cutoff identified in our analysis may therefore represent a pragmatic compromise when integrated with additional diagnostic parameters. While lesion size alone provided good discrimination, incorporating readily available clinical and biochemical parameters further improved malignancy prediction, suggesting that risk assessment may benefit from a combined rather than size-based approach alone.

Focusing specifically on small testicular masses—the group most likely to benefit from frozen-section-guided or testis-sparing surgery—we observed malignancy rates of 32.9% for lesions ≤5 mm and 44.4% for lesions ≤10 mm. Moreover, tumor size remained associated with lymphovascular invasion even within this subgroup. These findings highlight that small lesions still carry a clinically relevant risk of malignancy and that surgical decision-making should not be based on size alone.

Overall, our results indicate that tumor size represents a biologically and clinically relevant factor for risk stratification in testicular neoplasms within a real-world cohort. However, its utility is maximized when combined with ultrasound features, serum tumor markers, and patient-specific risk factors. Conservative management may be appropriate in carefully selected patients with small, well-characterized lesions, while larger or high-risk tumors warrant prompt surgical intervention.

The clinical role of contralateral testicular biopsy remains controversial [22,26]. While the European Association of Urology recommends biopsy in high-risk patients, routine biopsy is not universally endorsed due to the relatively low prevalence of germ cell neoplasia in situ, the risk of false-negative results, and concerns regarding overtreatment. In our cohort, contralateral testicular biopsy was performed in 64.3% of patients and detected GCNIS in 2.8%, which is relatively consistent with previously reported prevalence rates of 4–8% [27,28,29]. The distribution of GCNIS across histological subtypes—four cases in non-seminomatous and three in seminomatous GCTs—reflects the established risk across both major tumor categories [17,27]. Contralateral biopsy in our cohort was performed selectively based on guideline recommendations and individual clinical risk factors. Accordingly, the relatively high proportion of patients undergoing biopsy and the observed GCNIS prevalence should be interpreted in the context of this selection process and may not be representative of an unselected GCT population.

Preoperative serum tumor markers (LDH, AFP, and β-HCG) were largely within normal or mildly elevated ranges and showed no statistically significant associations with contralateral biopsy positivity. Although LDH showed a non-significant trend, this finding should be interpreted cautiously. These observations are in line with existing literature indicating that serum tumor markers have limited value in predicting GCNIS and are primarily used for staging and follow-up rather than GCNIS risk stratification [2,30]. Tumor size, pathological T stage, lymphovascular invasion, vascular invasion, and rete testis infiltration were likewise not associated with contralateral positivity, reinforcing evidence that established clinical risk factors—such as testicular atrophy or a history of cryptorchidism—are more informative predictors [20,22,27,30]. In multivariable penalized regression analysis, neither primary tumor size nor preoperative serum tumor markers independently predicted contralateral biopsy positivity. These findings suggest that contralateral GCNIS cannot be reliably inferred from characteristics of the index tumor or its biochemical activity. Given the low prevalence of contralateral positivity and the absence of robust tumor-related predictors, our results support current guideline recommendations favoring a selective approach to contralateral biopsy based on established clinical risk factors rather than primary tumor features alone.

This study has several limitations. Its retrospective design introduces potential selection bias, and the moderate sample size may limit statistical power for subgroup analyses, particularly for rare events such as GCNIS positivity. Furthermore, ultrasound measurements were obtained during routine clinical care by different operators, and interobserver variability could not be formally assessed. This is particularly relevant when interpreting specific lesion-size thresholds, as small differences in measurement may result in classification on either side of a proposed cutoff. Finally, the identified size cutoffs have not been prospectively validated, and their clinical applicability requires confirmation in larger, multicenter prospective studies.

## 5. Conclusions

The present study demonstrates that lesion size is a strong predictor of malignancy in testicular masses and correlates with established germ cell tumor risk factors. In the present cohort, a size threshold of 12.5 mm provided the most balanced discrimination between malignant and benign lesions, although bootstrap validation demonstrated uncertainty regarding the exact optimal threshold. Nevertheless, clinical decision-making should integrate additional imaging findings, serum tumor markers, and patient-specific risk factors rather than lesion size alone. Tumor size, histopathology, and serum markers were not associated with contralateral biopsy positivity, supporting a selective biopsy approach based primarily on established clinical risk factors.

## Figures and Tables

**Table 1:** Baseline characteristics of the study cohort.

Variable	Overall (*n* = 387)
**Age, years (median [IQR])**	37 [30–46]
**Histology,** ***n*** **(%)**	
Benign	103 (26.6)
Seminomatous GCTs	196 (50.6)
Non-seminomatous GCTs	87 (22.5)
GCNIS only	1 (0.3)
**Pathologic T stage,** ***n*** **(%)**	
pT1	225 (79.2)
pT2	39 (13.7)
pT3	19 (6.7)
pT4	1 (0.4)
**Lymphatic invasion,** ***n*** **(%)**	
Absent	257 (90.5)
Present	27 (9.5)
**Vascular invasion,** ***n*** **(%)**	
Absent	252 (88.7)
Present	32 (11.3)
**Rete testis invasion,** ***n*** **(%)**	
Absent	185 (65.1)
Present	99 (34.9)
**Margin status,** ***n*** **(%)**	
Negative	278 (97.9)
Positive	6 (2.1)
**Lymph node involvement,** ***n*** **(%)**	
Absent	245 (86.3)
Present	39 (13.7)
**Metastatic disease,** ***n*** **(%)**	
Absent	279 (98.2)
Present	5 (1.8)
**Serum tumor markers, median [IQR]**	
hCG (IU/L)	2.0 [2.0–3.5]
LDH (U/L)	214 [185–254]
AFP (µg/L)	2.8 [1.9–5.1]
**Contralateral biopsy positive,** ***n*** **(% of those biopsied)**	
Negative	242 (97.2)
Positive	7 (2.8)

**Table 2:** ROC Analysis Results of Different Clinical Size Cutoffs of Testicular Tumors.

Lesion Size Threshold (mm)	Sensitivity	Specificity	PPV	NPV	Accuracy	AUC	95% CI for AUC
**5**	0.95	0.38	0.81	0.72	0.80	0.83	0.78–0.87
**8**	0.87	0.60	0.86	0.62	0.80	0.83	0.78–0.87
**10**	0.84	0.61	0.86	0.57	0.78	0.83	0.78–0.87
**15**	0.73	0.76	0.89	0.50	0.74	0.83	0.78–0.87

## Data Availability

The data presented in this study are available on request from the corresponding author.

## Supplementary Materials

The following supporting information can be downloaded at: https://www.mdpi.com/article/10.3390/cancers18182907/s1. Supplementary Figure S1: Size distribution of benign and malignant lesions measuring ≤10 mm. Lesions were grouped into 1-mm size intervals according to maximum lesion diameter. Bars indicate the number of benign and malignant lesions within each size interval. Supplementary Figure S2: Decision-curve analysis of lesion size for predicting malignant histology. Net benefit is shown across threshold probabilities for the continuous lesion-size model and the Youden-derived 12.5-mm cutoff, compared with treat-all and treat-none strategies. Supplementary Figure S3: Illustration of the absolute risk of malignancy depending on lesion size in different size bins. Supplementary Figure S4. Association between lesion size and predicted probability of malignant histology. The solid line represents the predicted probability of malignancy derived from logistic regression using a natural cubic spline for lesion size (3 degrees of freedom). The shaded area represents the corresponding 95% confidence interval. Supplementary Figure S5: Penalized multivariable logistic regression predicting contralateral biopsy positivity. Supplementary Table S1: Histopathological diagnoses of benign testicular lesions in the cohort. Supplementary Table S2: Absolute malignancy risk at different tumor size cutoffs. Supplementary Table S3: Penalized multivariable logistic regression for contralateral biopsy positivity.
