# Supplementary material for: Sizing up Testicular Masses: Clinical Implications of Lesion Size and Contralateral Biopsy
Source: Cancers (Basel). 2026 Sep 8;18(18):2907. doi: 10.3390/cancers18182907 (PMC13604235; doi:10.3390/cancers18182907)
Supplement: Supplementary file 1 [file cancers-18-02907-s001.zip › cancers-4513736-supplementary.pdf]

Supplementary Material

Sizing Up Testicular Masses: Clinical Implications of Lesion Size and Contralateral Biopsy

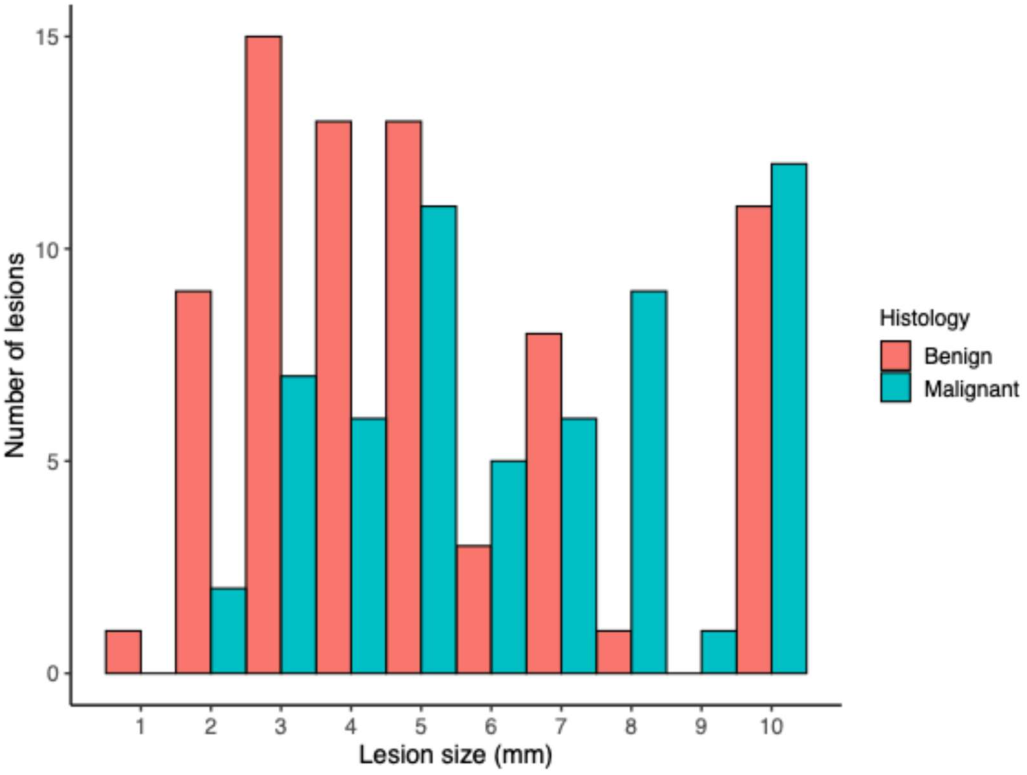

Supplementary Figure S1: Size distribution of benign and malignant lesions measuring ≤10 mm. Lesions were grouped into 1-mm size intervals according to maximum lesion diameter. Bars indicate the number of benign and malignant lesions within each size interval.

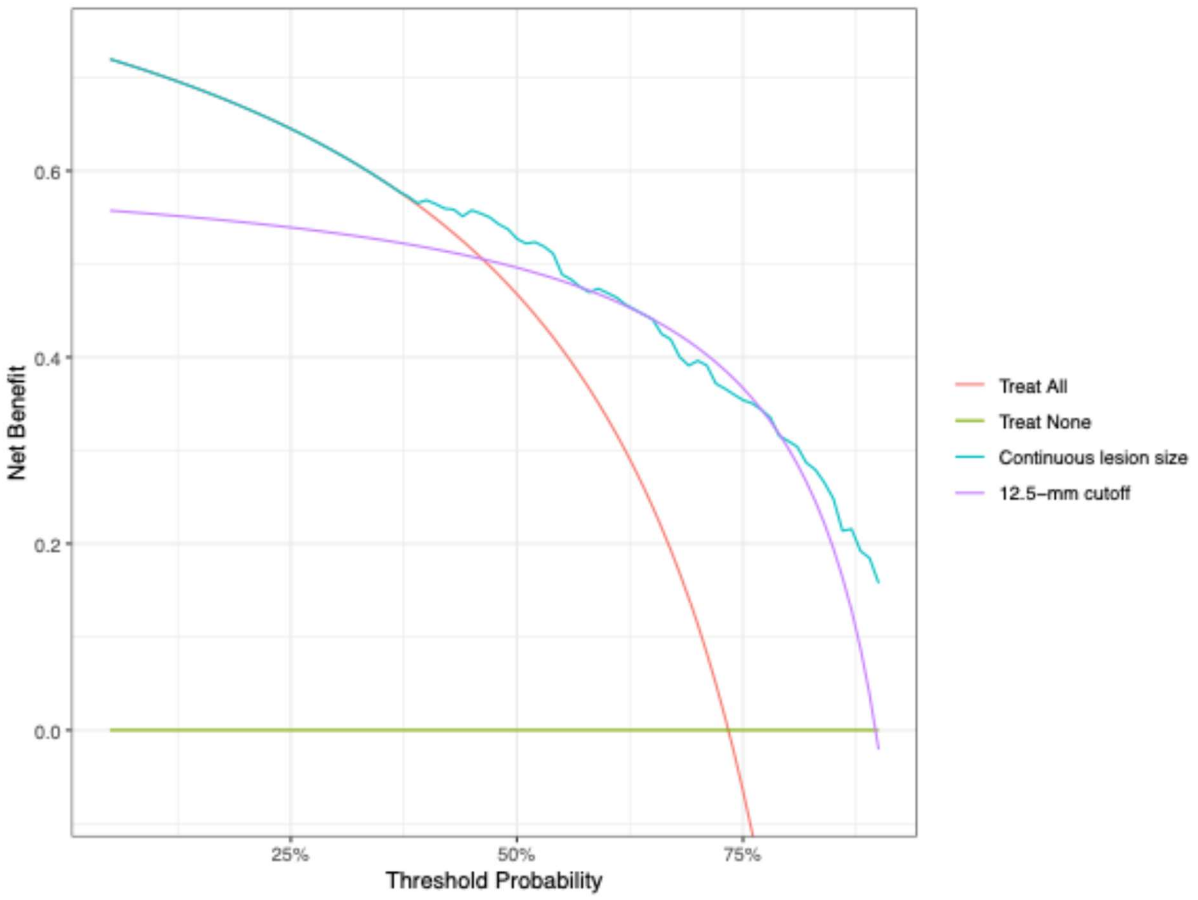

Supplementary Figure S2: Decision-curve analysis of lesion size for predicting malignant histology. Net benefit is shown across threshold probabilities for the continuous lesion-size model and the Youden-derived 12.5-mm cutoff, compared with treat-all and treat-none strategies.

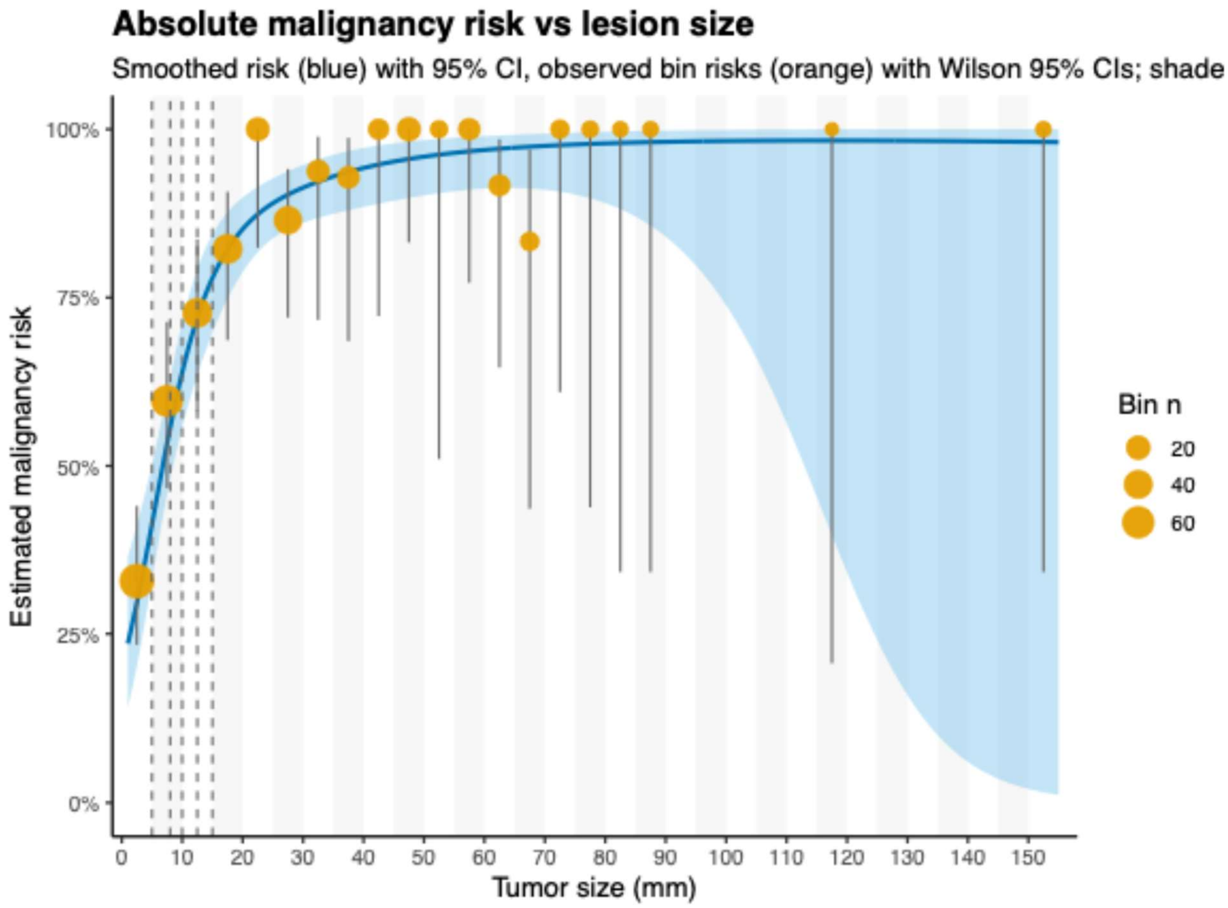

Supplementary Figure S3: Illustration of the absolute risk of malignancy depending on lesion size in different size bins.

Supplementary Table S1: Histopathological diagnoses of benign testicular lesions in the cohort.

Histopathological diagnosis	n
Benign Leydig cell tumour	32
Benign testicular tissue	18
Leydig cell hyperplasia	14
SCO (Sertoli cell-only syndrome)	7
Benign Sertoli cell tumour	7
Chronic inflammation	6
Epidermoid cyst	6
Adenomatoid tumour	4
Simple cyst	3
Haemangioma	2
Epitheloid granuloma	1
Fibrous pseudotumour	1
Intratesticular haematoma	1
Spermatocele	1

Supplementary Table S2: Absolute malignancy risk at different tumor size cutoffs.

Cutoff (mm)	Group	n	Malignant, n (%)	Risk (95% CI)
5	≤ 5 mm	76	25 (32.9)	23.4–44.1%
	> 5 mm	311	259 (83.3)	78.7–87.0%
8	≤ 8 mm	109	46 (42.2)	33.4–51.6%
	> 8 mm	278	238 (85.6)	81.0–89.3%
10	≤ 10 mm	133	59 (44.4)	36.2–52.8%
	> 10 mm	254	225 (88.6)	84.1–91.9%
12.5	≤ 12.5 mm	145	67 (46.2)	38.3–54.3%
	> 12.5 mm	242	217 (89.7)	85.2–92.9%
15	≤ 15 mm	177	91 (51.4)	44.1–58.7%
	> 15 mm	210	193 (91.9)	87.4–94.9%

Supplementary Table S3: Penalized multivariable logistic regression for contralateral biopsy positivity.

Variable	Odds Ratio (OR)	95% Confidence Interval	p-value
**Tumor size (per mm)**	1.03	0.99–1.08	0.128
**Age (per 10-year increase)**	0.47	0.14–1.20	0.122
**log(AFP)**	1.28	0.79–2.00	0.303
**log(β-HCG)**	0.76	0.39–1.26	0.304
**log(LDH)**	1.23	0.14–6.94	0.820

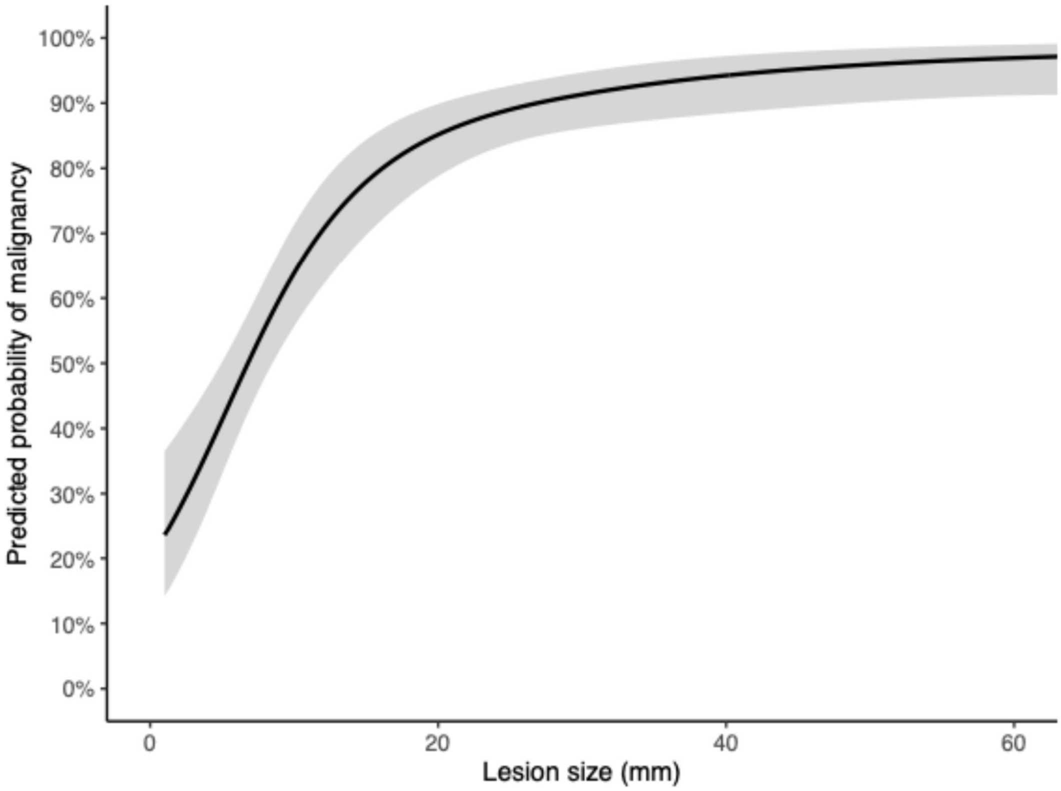

Supplementary Figure S4: Association between lesion size and predicted probability of malignant histology. The solid line represents the predicted probability of malignancy derived from logistic regression using a natural cubic spline for lesion size (3 degrees of freedom). The shaded area represents the corresponding 95% confidence interval.

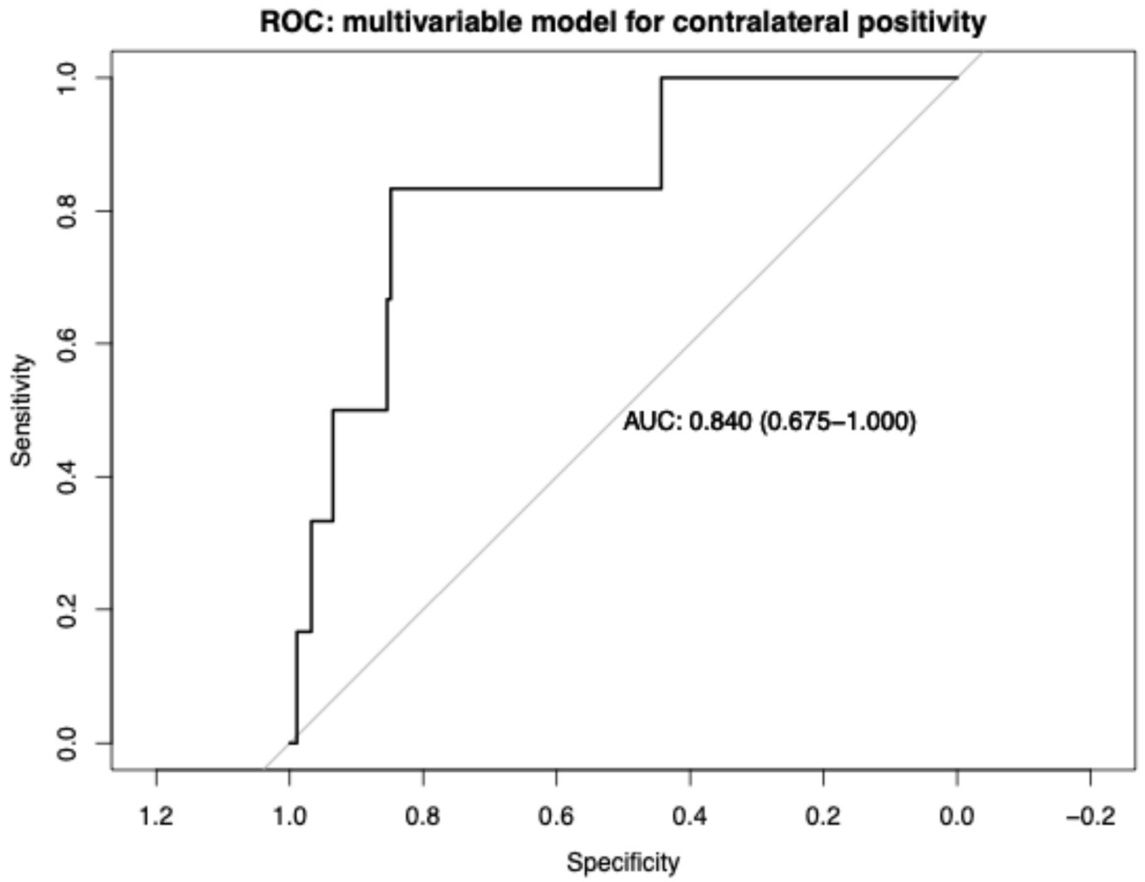

Supplementary Figure S5: Penalized multivariable logistic regression predicting contralateral biopsy positivity.
